# Cross-Cultural Analysis of Spiritual Bypass: A Comparison Between Spain and Honduras

**DOI:** 10.3389/fpsyg.2021.658739

**Published:** 2021-05-06

**Authors:** Alejandra Motiño, Jesús Saiz, Iván Sánchez-Iglesias, María Salazar, Tiffany J. Barsotti, Tamara L. Goldsby, Deepak Chopra, Paul J. Mills

**Affiliations:** ^1^Department of Social, Work and Differential Psychology, Complutense University of Madrid, Madrid, Spain; ^2^Department of Psychobiology and Behavioral Sciences Methods, Complutense University of Madrid, Madrid, Spain; ^3^Department of Family Medicine and Public Health, University of California, San Diego, San Diego, La Jolla, CA, United States

**Keywords:** spiritual bypass, cross-cultural analysis, spiritual well-being, stressful events, social support

## Abstract

Religion and spirituality (R/S) serve as coping mechanisms for circumstances that threaten people’s psychological well-being. However, using R/S inappropriately to deal with difficulties and problems in daily life may include the practice of Spiritual Bypass (SB). SB refers to avoiding addressing emotional problems and trauma, rather than healing and learning from them. On the other hand, coping strategies may be determined by the cultural context. This study aims to describe the presence of SB in individuals who may have experienced stressful situations and to understand the influence of culture on SB by comparing SB in two culturally different groups. The sample consists of a total of 435 people, 262 of Honduran nationality and 173 of Spanish nationality. Both groups are approximately equivalent in age and gender. The degree of SB, stressful events, perception of social support and spiritual well-being are examined, respectively, through the Spiritual Bypass Scale, and specific items and subscales from the Social Readjustment Rating Scale, Multidimensional Scale of Perceived Social Support, and the Functional Assessment of Chronic Illness Therapy - Spiritual Wellbeing. The results showed a higher spiritual well-being and use of SB in the Honduran sample as compared to the Spanish sample, but similar social support and stressful events. Furthermore, some of the factors predicting SB were different between the two samples. While age and a greater number of R/S practices were important in both samples, for the Honduran sample the variables that best explained SB were being a Christian, having greater social support, fewer stressful events, and greater attendance at church or temple. For the Spanish sample, however, the variable that best explained SB was studying R/S texts. Therefore, SB must be understood within the culture in which it develops, since in different cultural contexts it appears to relate to differing factors. Thus, SB becomes a possible functional or dysfunctional coping strategy depending on the social context.

## Introduction

According to information provided by the Pew Research Center: Hackett et al. (2018), it is estimated that 5.8 billion people identify with some religious affiliation. However, their geographical distribution varies considerably, such that, for example, in Honduras 90% of the population affirm that religion is important to them, while in Spain only 22% consider it that way.

Religions are phenomena created by human beings that manifest within a culture or society (Gomes, 2009). Thus, as part of a given culture, religions influence interpersonal and social processes (Anderson, 2015). At the same time, interpersonal and social factors influence how individuals acquire coping strategies, including certain religious coping strategies, which provide greater inner resources to face life events, help achieve a higher level of spiritual well-being, and experience feelings of closeness to God (Pargament et al., 1998a), as well as trust in people with whom they share the same beliefs (Diener et al., 2011).

Spirituality may be characterized as reaching beyond religion because it focuses on individuals’ experiences, unlike the former that attempts to explain these experiences within a particular belief system (Legere, 1984). Spirituality can arise within or outside of the religious context, providing people an opportunity to participate and connect to something perceived as greater than themselves (Forrest and Hunt, 1993). In this study, the concept of spirituality will be applied in a broad sense and identified with inner peace and meaning, while religiosity will refer to organized religious beliefs, practices, and participation. While the differences between the two terms (spirituality versus religion) are recognized, the present study will use the terms interchangeably when discussing their shared aspects.

Spiritual and religious (S/R) practices such as prayer, meditation, yoga, or reading religious writings have been argued to serve as a path to inner transformation (e.g., Welwood, 1984; Cashwell et al., 2007). The use of these practices may impact the decisions individuals make throughout the lifespan, especially those concerning the areas of health, quality of life, and coping strategies (Puchalski, 2012). Researchers have described spirituality and religiosity as important aspects of human development and positively related to well-being and health (e.g., Pargament et al., 1998a; Park and Edmondson, 2011; Anand et al., 2012; Koenig, 2012). However, there are also references to negative effects of S/R on health (Mohr et al., 2006) and, as described later, a few of these negative effects may be associated with spiritual bypass (SB), which in turn could also be closely mediated by cultural factors.

Given the centrality of religion as a coping mechanism in everyday life, the possibility exists that certain aspects of religious coping may be dysfunctional, as well as the possibility that cultural differences may exist with regard to religiosity. Thus, the current study sought to explore the possible appearance of SB during stressful events, compare it with other more adaptive resources (e.g., seeking social support), and examine the influence of the cultural context for the SB. We expect that these findings will significantly contribute to our understanding of the practice of SB and its cultural context.

### Spirituality and Religion as Coping Mechanisms

Researchers in the field of religion and spirituality have proposed that individuals seek to relieve stress through the search for and expression of the sacred or spirituality within their daily lives and in doing so, they may use their religion and spiritual beliefs as a coping mechanism (Pargament et al., 2011). A stressful situation may be defined as the perception that there is an imbalance between the capabilities of the individual and the demands of the environment (Abu-Raiya and Pargament, 2015). The mechanisms that underlie how spirituality and religion function as coping strategies remain under debate; however, religion may be a coping strategy that is an aspect of the larger societal cultural values system which offers a considerable variety of strategies to cope with life stressors (e.g., redefining the stressor through religion as benevolent and potentially beneficial, gaining control through a partnership with God, achieving comfort and reassurance through the love and care of congregation members as well as clergy, and so on). Thus, religion constitutes a path for providing meaning to certain life events and addressing critical situations (Pargament et al., 2005). As Kaplan (1976) also proposed, this state of well-being in times of crisis is possible because religion constitutes a support system for facing changes, growing personally, and discovering the purpose of life, all within a familiar and social context perceived as safe.

The use of religion as a coping mechanism by itself does not guarantee success in overcoming problems, as cultural factors may play a decisive role in that outcome (Pargament et al., 2005). In fact, when Sica et al. (1997) evaluated the cross-cultural properties of an instrument to measure coping strategies (COPE Inventory, Litman, 2006) when comparing an Italian sample with another North American sample, they discovered differences in an emotion-focused strategy described as using faith for support, e.g., turning to religion. On the other hand, certain types of religious coping methods are construed as counter-productive, due to the perception that they encourage avoidance of the problem rather than addressing it (e.g., blindly following a religious leader or waiting for a miracle to occur that solves a given problem) (Pargament et al., 1998b).

Finally, when spiritual practices are carried out collectively, the sense of belonging to that community is a predictive factor in the satisfaction and involvement the individual will experience with their spiritual beliefs (Pargament et al., 1983). Having a group of people with whom ideologies and interests are shared provides individuals a source of social support; therefore, their stress levels tend to reduce and greater life satisfaction is perceived (Tix and Frazier, 1998). In contrast, in congregations that are characterized by social restriction and authoritarian norms, there are lower levels of trust among their participants (Pargament et al., 1983). If individuals do not feel supported by their community environment, a spiritual struggle is generated which adversely affects psychological well-being and minimizes the perception of satisfaction (Park and Edmondson, 2011).

### Spiritual Bypass

Sigmund Freud (1901/1914) stated that religion was based in a projection of unconscious content, a means of avoiding the pain of reality. Although this was discussed by his own disciples (Jung, 1960), as well as other theorists (e.g., James, 1902/2009; Banks, 1973), part of Freud’s theory was the basis for the concept of Spiritual Bypass (SB). Welwood (1984) employed it for the first time to refer to the use of religion or spirituality as a coping mechanism that contributes to the repression of unresolved personal and emotional problems, instead of assisting individuals to overcome them.

Other researchers have made theoretical contributions that have enriched what was initially proposed by Welwood (1984). For example, Cashwell et al. (2010) distinguishes two manifestations of SB - spiritualization and psychological avoidance - and developed a scale to evaluate these concepts. Masters (2010) identified various types of magical thinking that may be present in a state of SB. These contributions are described below.

Individuals use SB as a strategy to avoid complicated emotions, experiences, or circumstances (Picciotto and Fox, 2017). This use of spiritual practices may correspond to the extrinsic use of religion that Allport and Ross (1967) explained, since it is not intended for the pursuit of a deeper search for meaning and instead is used for the purpose of personal gain. For this reason, it is believed that SB is a process that may damage the psychological well-being of an individual since it involves the utilization of spiritual life in a dysfunctional manner, generating a blockage in development, which may increase dysfunctional psychological symptoms (Cashwell et al., 2010). When this occurs, it may be necessary to address SB in the therapeutic context, with potential therapeutic techniques such as Motivational Interviewing (Clarke et al., 2013).

Other research on this topic hypothesizes that SB manifests in various ways, such as an external locus of control, denial of personal responsibility, spiritual obsessions, repression of psychological problems, and spiritual narcissism (Welwood, 2000; Cashwell et al., 2007). Additionally, SB may also occur when the individual’s spiritual beliefs and practices are not compatible with their daily life (Welwood, 2000).

#### Spiritualizing as a Coping Strategy

Spiritual bypass enables individuals to develop magical thinking and this type of thinking may manifest in various ways. An individual may have the conviction that he or she must face certain difficulties with the purpose of learning a spiritual lesson (Masters, 2010). On the other hand, magical thinking may consist of a mixture of superstitions and illusory connections that causes people to wait for divine intervention instead of acting themselves (Picciotto and Fox, 2017). This type of thinking releases the individual from any responsibility since their needs will be satisfied by divine intervention; the resulting “spiritualizing” is characterized as the use of magical thinking to explain unpleasant and pleasant situations.

Spiritualization is a common manifestation of SB, which occurs when people exaggerate the meaning of everyday activities or secular objects, attributing spiritual or sacred connotations, and exaggerating the positive aspects of life events while refusing to acknowledge the negative aspects of the circumstances or themselves (Picciotto and Fox, 2017). Thus, when faced with a situation that would typically be characterized as negative, people with high SB will justify it by stating that it is a spiritual test that they must overcome.

#### Psychological Avoidance as a Coping Strategy

Another manifestation of SB occurs when human beings do not accept the effect that their actions, beliefs and their surrounding environment have on their spiritual experience, therefore practicing psychological avoidance and becoming what Batson (1976) called “religious conformists.” In this case, identification with the dogmas of the religious institution to which people belong is rigid, uncritical, and dependent.

Closely related to this, one may distinguish between a religion of order (one that promotes conformism) and a liberating religion, in which practitioners are encouraged to grow and develop (Martín-Baró, 1989).

#### Consequences of Spiritual Bypass as a Coping Strategy

The use of religion to cope with negative events is usually more effective when it is used to promote improved spiritual well-being, rather than using it to prevent unpleasant situations from arising (Tix and Frazier, 1998). Thus, using SB as a coping strategy does not guarantee success when facing stressful situations.

Moreover, existing in a state of SB does not allow for spirituality growth, since individuals become estranged and disconnected from vital aspects of the human condition (Masters, 2010). Consequently, they may disconnect from investing time into caring for family members, maintaining interpersonal relationships, addressing emotions (both positive and negative), and other important aspects of daily life (Picciotto and Fox, 2017). This deprivation of their environment may present in a simple manner, such as disconnecting from a person who is not considered spiritual or be demonstrated in extreme situations such as the crusades, the inquisition, and the witch hunts, all under the guise of the promotion of the higher good and the search for the conversion of all (Batson, 1976).

Currently the most common problems that arise from SB tend to be compulsive kindness, repression of unwanted emotions, spiritual narcissism, extreme external locus of control, spiritual obsession, blind faith in charismatic leaders, renunciation of personal responsibility, and social isolation (Cashwell et al., 2007).

### Cultural Differences in Coping, Religion and Spirituality

“A social culture is an organized way of life which is based on a common tradition and conditioned by a common environment” (Dawson, 1948/2013, p.35), which in turn influences human interactions, including coping strategies. Through the socialization process, culture provides individuals with coping strategies for stressful situations (Aldwin, 2004). Due to cultural diversity, different cultures may favor varying strategies for coping with stress, ranging from “emotion-focused,” “external” or “indirect control” strategies to “problem-focused,” “internal control” or “direct approaches to mastery.” Cross-cultural studies have also confirmed that, in the case of adolescents, cultures also provide varying coping styles (e.g., Oláh, 1995).

Culture has also been defined as “a spiritual community which owes its unity to common beliefs and common ways of thought far more than to any uniformity of physical type (Dawson, 1948/2013, p. 36).” From this point of view, culture and religion influence each other (Loewenthal, 2013), as culture constitutes the basis for religion and religion is affected by a given culture’s social structure and common beliefs. As expressed by other authors (Burley, 2018), to gain a better understanding of why and how spiritual practices manifest, it is important to focus on the cultural environment in which they exist.

Religiosity and spirituality can be perceived through the lens of varied attitudes and values, as well as important existing differences between various religious affiliations (Pargament et al., 2005; Saiz et al., 2021a). Moreover, there may even be differences within the same religion when taking account of the cultural backgrounds of the practitioners (Cohen and Hill, 2007). A collectivist society is one in which its members feel strongly connected to the groups with which they identify. On the other hand, individualistic societies value individual independence (Markus and Kitayama, 1991). Following this approach, many American Protestant religious groups may be considered individualistic whereas other religious affiliations such as Hindus and Catholics are collectivistic (Cohen and Hill, 2007). In the former, all religious and spiritual experiences are viewed as a process that occurs uniquely between an individual and God, while in the latter, individuals are perceived as fundamentally connected with each other and their communities (Cohen and Hill, 2007).

An example of this concept may be demonstrated in the World Values Survey (WVS, 2020). In this survey, southern European countries such as Spain present average scores in the dichotomous dimensions of “Traditional values versus secular-rational values” and “Survival values versus self-expression values.” On the other hand, Central American countries such as Honduras score in the middle point in the dimension “Survival values versus self-expression values” but low in the dimension “Traditional values versus secular-rational values.” This implies that religious values are more important for Central American countries than for southern European countries, even though they traditionally derive from Christian-Catholic origins (Inglehart and Baker, 2000).

As discussed earlier in this paper, Spain and Honduras maintain a significant difference in regard to the percentage of the population that state religion is an important aspect of their lives. However, studies have also demonstrated additional important cultural differences between the two countries with respect to religion. For instance, Spain maintains individual secularization, a strong separation between Church and State, with younger individuals leaving the Catholic Church in large numbers (Pérez-Agote, 2010). In Honduras, on the other hand, religious practice tends to have a positive and significant influence on the lifestyle of young people (Castellanos-Hernández, 2011). Moreover, after recent natural disasters, rates of conversion to Evangelism have increased, as several Evangelical missions collaborated with the local population on the reconstruction of their community (Olivo-Enso, 2003). Comparing these two related yet distinct cultures might be useful in describing cultural influence on religious behaviors.

The current primary scientific literature on SB addresses this topic from a clinical perspective, delving into its the emotional impact on individuals who suffer from it; however, the influence of the cultural context on SB itself remains unknown. Thus, it is necessary to study SB during stressful events, to compare it with other more adaptive resources (e.g., seeking social support), and understand the influence of cultural context on SB.

The following study therefore aims to describe the presence of SB in individuals from the general population who may have experienced stressful situations and to characterize the influence of culture on SB, comparing the scores of SB of two culturally different groups (Honduras and Spain). In addition, the study aims to further evaluate the nature of SB as a coping strategy, as well as the relationship of SB to spiritual well-being and perceived social support. Additionally, number of stressful events related to loss, spiritual practices, and sociodemographic variables will be also analyzed.

Therefore, the following hypotheses are established:

H1: Given the cultural difference in both samples (Honduras and Spain), it is expected that the R/S practices will differ in the two cultural contexts, with Honduras’ amount of R/S practices higher.
H2: Considering the importance of the cultural context in the R/S itself (Loewenthal, 2013; Burley, 2018), it is expected that SB scores will be different in Honduras than in Spain, with Honduras demonstrating higher scores on SB.
H3: The appearance of SB will be greater in individuals who have suffered stressful events, have less social support and lower spiritual well-being, and who participate in a greater number of R/S activities.
H4: The explanatory factors of SB will be different in Honduras from that of Spain.

## Materials and Methods

### Design

In this exploratory study, since there was a large geographical distance between both samples, the evaluation instruments were administered through Google Forms. Prior to beginning the questionnaire, each participant read a brief introduction regarding the aim of the study in which they were also assured of their confidentiality and anonymity. Later, those who wished to continue with the research provided their informed consent. The survey’s average time for completion was approximately 10 min.

The study was approved by the Deontological Commission of the Faculty of Psychology of the Complutense University of Madrid with reference “2020/21_020.”

### Participants

The sample consisted of a total of 435 persons (70.1% women) between 15 and 76 years old (*M* = 36.4, *SD* = 13.1) of which 262 (60.2%) were Honduran and 173 (39.8%) were Spanish. These nationalities were chosen because despite sharing a common language and heritage, they exhibit important cultural differences, including the cultural values described in the WVS (2020).

Table 1 displays the characteristics of the total sample and the subsamples by nationality. When comparing the Honduran and Spanish subsamples, no differences were found in the proportion of men and women, *X*^2^ (1) = 0.08, *p* = 0.781 nor in the mean ages [Honduran (*M* = 35.7, *SD* = 13.3) and Spanish (*M* = 37.5, *SD* = 12.9), *t* (433) = −1.36, *p* = 0.174]. The selection of participants was carried out through a non-random strategic sampling, which consisted of contacting potential participants through social networks and those participants also sharing the questionnaires with other people. The first contacts were made in college settings in Spain and Honduras. The main inclusion criterion was to reside in the country of nationality.

**TABLE 1:** SBS-12 reliability (internal consistency).

**Factor**		**Estimate [CI 95%]**
Spiritual bypass (full scale)	α	0.949 [0.942,0.955]
	ω	0.953 [0.946,0.960]
Psychological avoidance	α	0.958 [0.951,0.965]
	ω	0.958 [0.95,0.966]
Spiritualizing	α	0.818 [0.762,0.855]
	ω	0.825 [0.777,0.857]

### Variables and Instruments

#### Spiritual Bypass Scale-12, Spanish Version (SBS-12)

The original scale (Spiritual Bypass Scale-13, SBS-13; Fox et al., 2017) identifies the qualities of SB and assesses how these qualities may influence the process of psycho-spiritual health and well-being. This instrument was only available in English, so it was necessary to translate it into Spanish. A fluent bilingual person whose native language was English then translated the items back into the original language to verify that the instrument’s semantics were not lost. The translated instrument was tested in a pilot sample. An item (”When someone confronts me, I tend to overanalyze his or her spiritual motivations for confronting me”) caused comprehension problems in this sample, so it was eliminated from the final version adapted to Spanish (see Appendix). The questionnaire was composed of 12 items and grouped into two dimensions: the first, psychological avoidance, contains 9 items and refers to avoiding complicated emotions or experiences through the individual’s spiritual beliefs (e.g., “When I feel emotional pain, the first thing I want to do is pray or meditate about it”). The second dimension, spiritualization, addresses the tendency to value daily activities as sacred as identified through three items (e.g., “When someone I know is experiencing hardship, I believe it is due to spiritual attacks or oppression”). The items are written on a Likert-type scale with four response options (from 0 = totally disagree, to 3 = totally agree). In this study, the final total score was used, which resulted from the sum of both dimensions, with higher values indicating greater SB. In the original version, it demonstrated a reliability of α = 0.85 for the total test, α = 0.82 for the psychological avoidance subscale and α = 0.75 for the spiritualization subscale. In this study, we show reliability indicators for the current sample, along with validity indicators (see section “Results”).

#### Stressful Life Events

The Social Readjustment Rating Scale (SRRS) was created by Holmes and Rahe (1967) and adapted to Spanish by González and Morera (1983). This scale contains a list of 61 life events that represent an alteration in people’s daily lives, such as “the death of the spouse,” “asking for a high-value mortgage” or “problems with the boss.” The participant must select the items that are appropriate to their situation from the last year and rate them from 1 to 100, according to their possible emotional reaction, impact, or stress reaction. For Holmes and Rahe (1967), an additional 200 or more stressful life events in one’s life increases the incidence of psychosomatic disorders. For the present work, in order to reduce the total number of items, we created a new variable for the number of stressful events from the SRRS, using the 9 items that implied losses (death of spouse, death of close family, death of friend, prolonged illness, illness of a family member, losing a job, changing jobs, divorce and separation). The participants were asked to answer whether they had suffered these events (Yes = 1, No = 0), to later add them without giving a weight to each one, so that the higher the score, the more stressful events suffered.

#### Multidimensional Scale of Perceived Social Support (MSPSS)

Social support has been considered an important source for the development of coping strategies (Greenglass, 1993), and in numerous studies, social support has been analyzed along with religious coping as a reliable variable for effective coping when dealing with stressful events (Prati and Pietrantoni, 2009). Thus, we use it in the present study to compare SB with this widely used strategy.

This scale was developed by Zimet et al. (1988) to assess the perception of social support in family relationships, friends, and significant others. The questionnaire has a total of 12 items written on a Likert-type scale with five answer options (from 0 = Nothing, to 4 = Very much). For this research, only the four items of the subscale “Other significant people” were used, taken from the adaptation to Spanish by Trejos-Herrera et al. (2018), using the average of the items as the final score. The present researchers decided to use this dimension since it allowed participants to respond in a broad way regarding their sources of support (e.g., “there is a special person who is close to me when I need it”). For the present study, this subdimension yielded a Cronbach’s alpha coefficient of α = 0.94.

#### Functional Assessment of Chronic Illness Therapy–Spiritual Wellbeing (FACIT-Sp-12)

This scale was designed by Peterman et al. (2002) to measure the spiritual well-being of individuals with various chronic clinical circumstances. However, the researchers in the present study chose to include it given the demonstrated sensitivity to assess spiritual well-being in mixed situations of health and illness (González-Sanguino et al., 2020; Saiz et al., 2020). The test has a total of 12 Likert-type items with five response options (from 0 = Not at all, to 4 = Very much); thus, the higher the score, the greater the spiritual well-being. This scale is divided into the subscales of “Meaning/Peace” and “Faith.” In order to maintain a reduced number of items, the first dimension, consisting of 8 items, was applied for this research, since it explores spiritual well-being regardless of the faith or creed professed (e.g., “I feel peaceful”). Thus, for this research, FACIT-Sp-12 was selected to explore “spirituality” specifically. The internal consistency of this subscale for the present study was α = 0.85.

#### Religious or Spiritual Practices

To determine the religious (and spiritual) involvement of our participants, they were asked to report if they were involved in any of the following R/S practices: prayer, meditation, yoga, attending church or temple, prayer or spiritual group, study of religious texts, or other spiritual activity. It was recorded as “Yes = 1, No = 2.” In addition, the number or practices were summed, potentially ranging from 0 to 7. Also, the participants reported the frequency with which they performed those practices (1 = Almost never or never, 2 = Less than once in a year, 3 = Once a year, 4 = Only on holy or special days, 5 = Only when I attend spiritual services, 6 = Several times a week, 7 = Once a day, and 8 = Several times a day).

### Analysis

The categorical variables were described for the full sample and by nationality using frequency and contingency tables (number and percent of cases), as well as chi-squared tests of independence to study the relationship between variables. When appropriate, Cramér’s *V* was used as a measure of association. The continuous variables were described, their means compared via *t*-tests, and *R*-squared as an estimate of the effect size, when required.

As we translated and adapted SBS-12 into Spanish, we assessed its psychometric properties. To evaluate the reliability, we estimated its internal consistency using α and ω (and their 95% CI). The correlation between SBS-12 and FACIT-Sp-12 scores was used as an indicator of convergent validity. To test factor validity, the scores obtained in the SBS-12 were used to confirm the theoretical subjacent structure through confirmatory factor analysis (CFA). We assessed multivariate normality of the items using Mardia’s multivariate kurtosis and skewness coefficients (Mardia, 1970). As the hypothesis of normality was rejected, we used a weighted least square mean and the variance adjusted (WLSMV) estimator, a robust estimation method for categorical data (Brown, 2015). Several fit indexes were employed: chi square statistic to degrees of freedom ratio (χ^2^/df), root mean square error of approximation (RMSEA) with its 90% confidence interval, standard root mean square residual (SRMR), comparative fit index (CFI), and Tucker-Lewis index (TLI). We compared the values with the thresholds recommended by Chau (1997) and Schreiber et al. (2006). We interpreted the magnitude, direction, and statistical significance of the standardized parameter estimates.

We carried out hierarchical regression analyses (forward method) for each subsample. Age, gender, religious affiliation, individual religious practices, practice frequency, MSPSS and FACIT-Sp-12 scores, and the number of stressful life events reported were used as predictor variables. For the regression models, the categorical variable ‘religious affiliation’ was transformed into two dummy variables to represent the change from no religion (no religion, agnostic, or atheist) to Christian, and from no religion to other (Muslim, Hindu, Buddhist, Jew, or other). Multicollinearity was checked via Variance Inflation Factor (VIF) for each predictor variable.

We used several R packages: The CFAs were carried out using Lavaan, Version 0.6-3 (Rosseel, 2012); estimators α and ω and their CI were computed using MBESS, Version 4.4.3 (Kelley, 2018); Mardia’s multivariate analysis via MVN (Korkmaz et al., 2014). The remaining statistical analyses were carried out using SPSS Version 25.

## Results

### Psychometric Properties of the SBS-12

#### Reliability

The SBS-12 displayed sufficient reliability via internal consistency (the total scale, as well as its subscales), as shown in Table 2.

**TABLE 2:** SBS-12 fit indices for second-order confirmatory factor analysis model.

		**Full sample (*n* = 435)**	**RV**
Fit index	χ^2^/*df*	2.158	≤ 3.000
	RMSEA	0.052	< 0.060 to 0.080
	RMSEA [90% CI]	[0.039,0.065]	< 0.060 to 0.080
	SRMR	0.059	≤ 0.080
	CFI	0.982	≥ 0.950
	TLI	0.978	≥ 0.950

*RV, recommended values (Chau, 1997; Schreiber et al., 2006).*

#### Validity

Some evidence of convergent validity was found; SBS-12 and FACIT-Sp-12 scores showed a positive, significant correlation, *r* = 0.205; both instruments shared 6.3% of their variance, *r*^2^ = 0.063.

**FIGURE 1:**
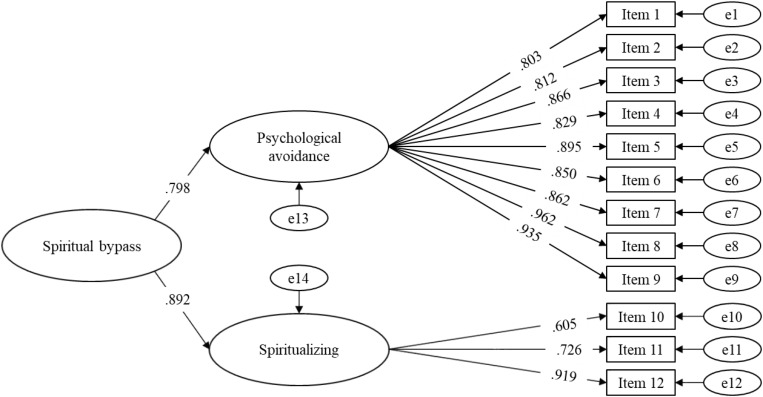
SBS-12 factor structure. Model of second-order factor. Standardized coefficients. All factor loadings are statistically significant (*p*s < 0.001), *N* = 435.

The CFA model demonstrated a good fit for all the considered indices (Table 3). Moreover, all the factor loadings were positive and significant for their assigned factor; from 0.803 to 0.962 for Psychological Avoidance, and from 0.606 to 0.919 for Spiritualizing (Figure 1), thus exceeding the values recommended by Brown (2015). Also, the second order factor Spiritual Bypass showed positive, significant factor loadings both for Psychological Avoidance (0.798) and Spiritualizing (0.892).

**TABLE 3:** Characteristics of participants, full sample by nationality. gender, affiliation, and R/S practices.

	**Full sample**		**Honduran**		**Spanish**					
**Variable**	***n***	**%**	***n***	**%**	***n***	**%**	***X*^2^**	***df***	***p***	***V***
Gender							0.08	1	0.781	–
Female	305	70.1	185	70.6	120	69.4				
Male	130	29.9	77	29.4	53	30.6				
Affiliation							102.2	2	<0.001	0.436
Christian	358	82.3	**255**	**97.3**	103	59.5				
Other*	9	2.1	1	0.4	**8**	**4.6**				
None**	68	15.6	6	2.3	**62**	**35.8**				
Prayer							173.2	1	<0.001	0.631
Yes	286	65.7	**236**	**90.1**	50	28.9				
No	149	34.3	26	9.9	**123**	**71.1**				
Meditation							12.01	1	0.001	0.166
Yes	103	23.7	47	17.9	**56**	**32.4**				
No	332	76.3	**215**	**82.1**	117	67.6				
Yoga							49.9	1	<0.001	0.339
Yes	57	13.1	10	3.8	**47**	**27.2**				
No	378	86.9	**252**	**96.2**	126	72.8				
Going to church/temple							75.74	1	<0.001	0.414
Yes	201	46.2	**165**	**63.0**	36	20.8				
No	234	53.8	97	37.0	**137**	**79.2**				
Prayer/spiritual group							4.02	1	0.045	0.096
Yes	6	1.4	**6**	**2.3**	0	0.0				
No	429	98.6	256	97.7	**173**	**100.0**				
Study of texts							49.22	1	<0.001	0.336
Yes	154	35.4	**127**	**48.5**	27	15.6				
No	281	64.6	135	51.5	**146**	**84.4**				
Other							0.15	1	0.700	–
Yes	17	3.9	11	4.2	6	3.5				
No	418	96.1	251	95.8	167	96.5				
Practice Frequency							147.10	7	< 0.001	0.581
Almost never or never	76	17.5	4	1.5	**72**	**41.6**				
Less than once in a year	7	1.6	2	0.8	5	2.9				
Once a year	8	1.8	2	0.8	**6**	**3.5**				
Only on holy or special days	20	4.6	11	4.2	9	5.2				
Only when I attend spiritual services	23	5.3	15	5.7	8	4.6				
Several times a week	108	24.8	67	25.6	41	23.7				
Once a day	100	23.0	**82**	**31.3**	18	10.4				
Several times a day	93	21.4	**79**	**30.2**	14	8.1				

V = Cramér’s measure of association. In bold type, cell counts and percentages higher than expected by the null hypothesis of independence. *Muslim, Hindu, Buddhist, Jew, and other. **No religion, agnostic, or atheist.

### Differences by Nationality in R/S Practices and Psychometric Instruments Used

Participant characteristics may be viewed in Table 1, as well as the specific practices participants observed. 97.3% of the Honduran participants considered themselves as Christians, versus the 59.5% of the Spanish participants. The total number of religious or spiritual practices ranged from 0 to 6 (*M* = 1.9, *SD* = 1.3). Consistent with the first hypothesis, we found significant differences in the R/S practices between both nationalities. The Honduran participants are involved in more religious practices (*M* = 2.3, *SD* = 1.1) than the Spanish participants (*M* = 1.3, *SD* = 1.2), *t* (433) = 8.90, *p* < 0.001, *r*^2^ = 0.154.

Specifically, in the Honduran sample there was a greater proportion of participants who pray, visit their church or temple, participate in more prayers or spiritual groups, and study more R/S texts. Furthermore, the frequency of religious practices in the Honduran sample is “once a day,” while in the Spanish sample it is “almost never or never.” In contrast, the Spanish sample practices more meditation and yoga than the Honduran sample.

Consistent with the second hypothesis, as may be viewed in Table 4, we found significant differences in SB between the nationalities, with the Honduran sample having the highest scores. In relation to the other psychometric tests, we found that the Honduran sample also demonstrates greater spiritual well-being (FACIT-Sp-12). Additionally, there were no significant differences in the amount of stressful life events or perceived social support (MSPSS) between the Honduran sample and the Spanish sample.

**TABLE 4:** Descriptive statistics of measurement instruments, and comparisons by nationality.

	**Full sample**			**Honduran**			**Spanish**						
**Variable**	***n***	***M***	***SD***	***n***	***M***	***SD***	***n***	***M***	***SD***	***t***	***df***	***p***	***r*^2^**
SBS-12	435	17.3	9.3	262	22.4	6.7	173	9.6	7.1	18.94	433	<0.001	0.453
Stressful life events	435	1.2	1.1	262	1.3	1.1	173	1.2	1.1	0.78	433	0.434	–
MSPSS	433	3.4	0.9	260	3.4	0.9	173	3.4	0.8	0.18	431	0.861	–
FACIT-Sp-12	433	24.5	5.4	260	25.0	5.3	173	23.8	5.5	2.16	431	0.031	0.011

*SBS-12, spiritual bypass scale-12 (Spanish 12 items version); MSPSS, multidimensional scale of perceived social support; FACIT-Sp-12, functional assessment of chronic illness therapy - spiritual wellbeing.*

### Predictive Models of Spiritual Bypass by Country

Contrary to what was stated in the third hypothesis, the regression analyzes included in Table 5 revealed that for the Honduran sample, two of the six variables that best explain SB (35.4% of the variance) are perceived social support and lower stressful events. In addition, age (the older the more SB), performing a greater number of R/S practices, being a Christian, and attending church or temple were also relevant variables to explain SB.

**TABLE 5:** SBS-12 regression models, by nationality.

**Variable**	***B***	**95% CI for *B***		***SE B***	**β**	***R*^2^**
		**LL**	**UL**			
**Honduran sample**						0.354
Constant	−0.91	−6.98	5.15	3.08	–	
Number or practices	1.57***	1.05	2.09	0.26	0.32	
Catholic	9.69***	5.34	14.03	2.21	0.23	
Age	0.09**	0.03	0.14	0.03	0.17	
MSPSS	1.03**	0.31	1.75	0.37	0.14	
Stressful life events	−0.79*	−1.38	−0.19	0.30	−0.13	
Going to church/temple	−1.62*	−3.04	−0.19	0.72	−0.12	
**Spanish sample**						0.427
Constant	7.96**	2.46	13.46	2.78	–	
Number or practices	1.47***	1.14	1.79	0.17	0.55	
Age	0.08*	0.01	0.14	0.03	0.14	
Study of texts	−3.67**	−6.07	−1.28	1.21	−0.19	

MSPSS, multidimensional scale of perceived social support. Forward regression method, final models. CI, confidence interval; LL, lower limit; UL, upper limit. *p < 0.050. **p < 0.010. ***p < 0.001.

As established in the fourth hypothesis, in the Spanish sample, the variables that best explain SB (42.7% of the variance) differ from those of the Honduran sample. Only three variables were significant, including two of the variables also important for the Honduran sample (performing a greater number of R/S practices and age), as well as a new variable, studying R/S texts.

Multicollinearity was not considered an issue, as no predictor variable showed a VIF greater than 2.4.

## Discussion

The present study examined the presence of SB in individuals from the general population who may have experienced stressful situations, and compared the scores and meaning of SB in two culturally different groups.

We observed significant differences between the two samples. Several of these differences relate to the frequency and type of R/S practices of the participants. Individuals who reside in countries with stable and developed economies tend to have lower levels of religiosity, unlike those who live in countries with high rates of poverty, insecurity and disease, whose population strongly identifies with religion (Diener et al., 2011). This coincides with the results of our study, in which the Honduran sample reported higher levels of identification with a (Christian) religion and engaged in more R/S practices. In addition, when comparing the results of the psychometric tests used in both samples, we observed that the Honduran sample reported more SB and spiritual well-being, while both samples showed very similar scores in perceived social support and in stressful life events experienced.

Regarding the explanation of SB usage, we found that for both samples, older age and performing religious activities such as attending church or temple (in Honduras) or studying religious texts (in Spain) explained the phenomenon. As had been hypothesized, SB was experienced by those individuals who previously found themselves immersed in practices and contexts related to R/S. However, it is striking that for the Honduran sample, SB was also explained by maintaining social support and by experiencing a lower number of stressful life events. This, in line with the higher spiritual well-being scores found in the Honduran sample, may mean that the SB has a different meaning for the Honduran sample, far from the negative connotations that it seems to have in other studies (Welwood, 2000; Cashwell et al., 2007). Also, this could indicate that the Honduran sample achieved a certain well-being using the SB as a coping strategy. Finally, experiencing a higher level of social support may be another cultural element that allowed the Honduran sample to interpret stressful events that they experienced as minor.

Several potential study limitations should be pointed out. In this study population we did not distinguish between different groups of Christians (Catholics, Protestants, Evangelists, etc.), which may be an important variable in the explanation of SB. The stressful events evaluation strategy was quantitative, and even though it reported the number of situations that the individual experienced, it did not allow for knowing the intensity nor the meaning attributed to the events. It is possible that other qualitative research strategies would enable a more in-depth ability to characterize the degree to which individuals are affected by these events, as well as other resources used by individuals to manage the situation. Another variable that could be promising in the explanation of SB for future research is to examine the intrinsic or extrinsic motivation of individuals to carry out R/S practices (Allport and Ross, 1967), since it is possible that SB is mediated by religious motivations; this might partially explain certain differences here attributed to culture. Accordingly, as our results are based on spirituality (peace and meaning) scores and R/S practices, it may be necessary to differentiate further between spirituality and religiosity, considering other variables and instruments to examine the degree of religiosity of an individual (Huber and Huber, 2012) and their spiritual needs (Büssing et al., 2018). Finally, we had predominantly Christian participants and it would be important to evaluate the effects of other religions on SB.

Several authors support introducing the R/S dimension regarding individuals with various psychosocial problems (Turbott, 1996; Saiz et al., 2021b), and addressing the cultural context of the of the individuals. In fact, we observed a striking relationship between number of stressful life events, less social support, and lower levels of emotional well-being for those in socially vulnerability situations, such as homeless individuals (Roca et al., 2019) and those with a diagnosis of mental illness (López and Laviana, 2007).

A recommendation to include R/S practice as a strategy to promote spiritual well-being should include measurement of the use of SB as a dysfunctional coping strategy. In caring for groups at risk of social exclusion, the cultural variable acquires fundamental importance in understanding the meaning attributed to experiences of suffering and emotional distress, as well as in assessing and enhancing coping strategies (culturally mediated) that may be useful in support and recovery processes (e.g., R/S practices, sense of belonging to socially valued groups or use of mutual support networks).

Finally, given that the relationship between religion and country-specific processes is likely bidirectional and culturally evolving (Cohen and Hill, 2007), and bearing in mind the importance of R/S for individual health and well-being, a cultural approach to SB should be considered to facilitate the well-being of individuals who face various stressful life events.

## Data Availability Statement

The raw data supporting the conclusions of this article will be made available by the authors, without undue reservation.

## Ethics Statement

The studies involving human participants were reviewed and approved by Deontological Commission of the Faculty of Psychology of the Complutense University of Madrid with reference “2020/21_020”. The patients/participants provided their written informed consent to participate in this study.

## Author Contributions

AM, JS, TB, and PM: conceptualization. IS-I and JS: methodology and formal analysis. IS-I: software. IS-I and AM: validation. AM and JS: investigation and resources. AM, IS-I, and JS: data curation. JS, AM, MS, and TG: writing–original draft preparation. AM, JS, MS, PM, TB, DC, and TG: writing–review and editing. JS: visualization and project administration. JS and PM: supervision and funding acquisition. All authors have read and agreed to the published version of the manuscript.

## Conflict of Interest

The authors declare that the research was conducted in the absence of any commercial or financial relationships that could be construed as a potential conflict of interest.

## Appendix

The 12 items used from the spiritual bypass scale.

**Table:**

**Spiritual Bypass Scale (Original)**	**Spiritual Bypass Scale (Spanish version)**
1) My spiritual life helps me feel my emotions more fully.	1) Mi vida espiritual me ayuda a sentir mis emociones de forma más plena.
2) When I feel emotional pain, the first thing I want to do is pray or meditate about it.	2) Cuando siento dolor emocional, lo primero que quiero hacer es orar o meditar al respecto.
3) When I am in pain, I believe God will deliver me from it.	3) Cuando estoy en dolor, creo que una fuerza superior me librará de eso.
4) When something tragic happens (to me or to others) I say that God will intervene.	4) Cuando algo trágico ocurre (a mi o a otros) digo que Dios intervendrá.
5) It is more important to me to seek spiritual guidance than to seek aid from a psychological helper.	5) Es más importante para mí buscar guía espiritual que buscar la ayuda de un psicólogo.
6) When experiencing difficulties, I believe it is most important to deal with the spiritual source of my problems.	6) Cuando experimento dificultades, creo que es más importante lidiar con la fuente espiritual de mis problemas.
7) I believe it is preferable to cure emotional problems by being spiritually advanced.	7) Creo que es preferible curar los problemas emocionales siendo más avanzados espiritualmente.
8) It is more important for me to be spiritually awakened than to feel emotionally intact.	8) Es más importante para mí tener un despertar espiritual que sentirme emocionalmente intacto.
9) I believe that healing one’s spirit takes precedence over healing their emotions.	9) Creo que sanar el espíritu es preferente a sanar la emociones.
10) When someone I know is in trouble, I believe it is because they have done something wrong spiritually.	10) Cuando alguien que conozco tiene problemas, creo que es porque ha hecho algo mal espiritualmente.
11) When someone I know is experiencing hardship, I believe that it is due to spiritual attack/oppression.	11) Cuando alguien que conozco experimenta dificultades, creo que es debido a ataques u opresiones espirituales.
12) When I face a life challenge, I always consult with a spiritual or religious teacher.	12) Cuando me enfrento a un desafío de la vida, siempre consulto con un maestro espiritual o guía religioso.
